# Human apolipoprotein E glycosylation and sialylation: from structure to function

**DOI:** 10.3389/fnmol.2024.1399965

**Published:** 2024-08-07

**Authors:** Hee-Jung Moon, Yan Luo, Diksha Chugh, Liqin Zhao

**Affiliations:** ^1^Department of Pharmacology and Toxicology, School of Pharmacy, University of Kansas, Lawrence, KS, United States; ^2^Neuroscience Graduate Program, University of Kansas, Lawrence, KS, United States

**Keywords:** apolipoprotein E (ApoE), posttranslational modification (PTM), glycosylation, sialylation, Alzheimer’s disease

## Abstract

Human apolipoprotein E (ApoE) was first identified as a polymorphic gene in the 1970s; however, the genetic association of ApoE genotypes with late-onset sporadic Alzheimer’s disease (sAD) was only discovered 20 years later. Since then, intensive research has been undertaken to understand the molecular effects of ApoE in the development of sAD. Despite three decades’ worth of effort and over 10,000 papers published, the greatest mystery in the ApoE field remains: human ApoE isoforms differ by only one or two amino acid residues; what is responsible for their significantly distinct roles in the etiology of sAD, with ApoE4 conferring the greatest genetic risk for sAD whereas ApoE2 providing exceptional neuroprotection against sAD. Emerging research starts to point to a novel and compelling hypothesis that the sialoglycans posttranslationally appended to human ApoE may serve as a critical structural modifier that alters the biology of ApoE, leading to the opposing impacts of ApoE isoforms on sAD and likely in the peripheral systems as well. ApoE has been shown to be posttranslationally glycosylated in a species-, tissue-, and cell-specific manner. Human ApoE, particularly in brain tissue and cerebrospinal fluid (CSF), is highly glycosylated, and the glycan chains are exclusively attached via an *O*-linkage to serine or threonine residues. Moreover, studies have indicated that human ApoE glycans undergo sialic acid modification or sialylation, a structural alteration found to be more prominent in ApoE derived from the brain and CSF than plasma. However, whether the sialylation modification of human ApoE has a biological role is largely unexplored. Our group recently first reported that the three major isoforms of human ApoE in the brain undergo varying degrees of sialylation, with ApoE2 exhibiting the most abundant sialic acid modification, whereas ApoE4 is the least sialylated. Our findings further indicate that the sialic acid moiety on human ApoE glycans may serve as a critical modulator of the interaction of ApoE with amyloid β (Aβ) and downstream Aβ pathogenesis, a prominent pathologic feature in AD. In this review, we seek to provide a comprehensive summary of this exciting and rapidly evolving area of ApoE research, including the current state of knowledge and opportunities for future exploration.

## 1 Introduction

**Figure 1:**
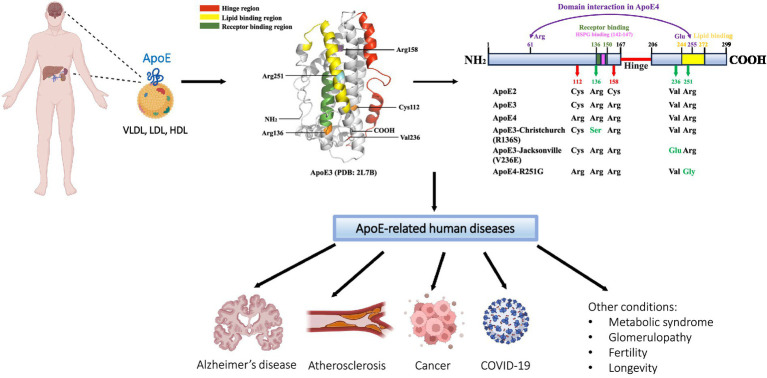
Schematic of ApoE protein source, structure, and related diseases. ApoE is primarily synthesized and secreted from the liver and brain; other tissues of ApoE production include the kidney, endometrium, skin, adrenal gland, and reproductive tissues. As a significant component of VLDL, LDL, and HDL lipoprotein particles, ApoE functions as a major regulator of lipid transport and metabolism. Human ApoE exists in three common isoforms, ApoE2, ApoE3, and ApoE4, which differ by one or two amino acids at positions 112 and 158. Several rare variants of ApoE have recently been found to modulate the risk for the development of Alzheimer’s disease (AD). In addition to AD, ApoE has been linked to a number of other human diseases, including cardiovascular disease, infectious diseases such as COVID-19, cancer, and others. The ApoE3 three-dimensional structural model is retrieved from the RCSB Protein Data Bank (PDB: 2L7B); specific regions and residues are highlighted using PyMOL 2.5.7.

Apolipoprotein E (ApoE), encoded by the *APOE* gene, is a 34 kDa, 299-amino-acid protein that was first isolated and identified as a component of plasma very low-density lipoproteins (VLDL) (Utermann, 1975). The human *APOE* gene is located on chromosome 19, consisting of four exons separated by three introns and spanning 3,597 bp (Olaisen et al., 1982; Das et al., 1985; Paik et al., 1985). The *APOE* gene generates five mRNA transcripts, with four being protein-coding. The full-length transcript (*APOE-201*) translates to a pre-ApoE isoprotein of 317-amino-acid, including an N-terminal 18-amino-acid signal peptide. Subsequently, the pre-ApoE isoprotein undergoes proteolytic processing and posttranslational modification (PTM) to produce the mature ApoE protein (Rall et al., 1982b; Zannis et al., 1984). The other three protein-coding truncated transcripts (*APOE-202*, *APOE-203*, and *APOE-204*) give rise to ApoE fragments of variable lengths (219aa, 269aa, and 216aa, respectively) (e!Ensembl, 2024). The full-length mature ApoE protein consists of two functional domains, namely N- and C-terminal domains, which are connected by a hinge region. The N-terminal domain (residues 1–167) forms an anti-parallel four-helix bundle that contains a highly positively charged receptor-binding region (residues 136–150). The C-terminal domain (residues 206–299) includes a lipid-binding region (residues 244–272) (Bradley et al., 1982; Dong et al., 1994; Weisgraber, 1994) (Figure 1). In the periphery, the liver is the primary source of ApoE, contributing to approximately 75% of the total pool of peripheral ApoE. In addition, ApoE is produced in the adrenal gland, kidney, testis, endometrium, placenta, skin, and bone marrow (Blue et al., 1983; Atlas, 2024). ApoE expression in the brain is similarly heterogeneous. Although astrocytes are known to be the primary source of ApoE production and secretion, followed by microglia (Uchihara et al., 1995; Achariyar et al., 2016). ApoE is also *de novo* synthesized in neurons, albeit at lower levels than glial cells. It is reported that the neuronal source of ApoE represents approximately 20% of total ApoE in the cortex (Xu et al., 2006; Knoferle et al., 2014). Although the exact role of ApoE in neurons is not fully understood, the markedly upregulated expression of ApoE observed in injured or stressed neurons may indicate a potentially important role of neuronal ApoE in neuronal repair mechanisms (Boschert et al., 1999; Aoki et al., 2003; Theendakara et al., 2016). As a member of lipid-binding apolipoproteins, ApoE plays an essential role in the metabolism of triglyceride-rich lipoproteins (Mahley, 1988). ApoE-containing lipoproteins are initially bound to cell-surface heparan sulfate proteoglycans (HSPG) and then interact with LDL receptor (LDLR) for endocytosis (Ji et al., 1993, 1994). Moreover, ApoE has been shown to regulate the deposition or clearance of cerebral and systemic amyloid plaques (Wisniewski and Frangione, 1992; Näslund et al., 1995). ApoE was first identified to be a major risk factor for atherosclerosis (Mahley, 1988). Today, ApoE has been associated with various diseases, including Alzheimer’s disease (AD), infectious diseases such as Covid-19, cancer, metabolic syndrome, glomerulopathy, fertility, and longevity (Mahley, 1988; Martínez-Martínez et al., 2020; Saito et al., 2020; Yu et al., 2022; Zhang et al., 2022) (Figure 1). However, the exact role of ApoE in the pathogenesis of these diseases remains largely unknown.

## 2 ApoE major isoforms

The genetic polymorphism of human ApoE was discovered in 1977 (Utermann et al., 1977). The human *APOE* gene encodes for three primary allelic isoforms: ε2, ε3, and ε4, which contribute to three homozygous (*APOE2/2*, *APOE3/3*, and *APOE4/4*) and three heterozygous (*APOE3/2*, *APOE4/2*, and *APOE4/3*) genotypes (Das et al., 1985; Weisgraber, 1994). *APOE3* represents the most common allele and is found in 77.9% of the general population, followed by the approximate 13.7% frequency for *APOE4*, and 8.4% for *APOE2* (Farrer et al., 1997). There are only one or two amino acids that differ at positions 112 and 158 among the three isoforms, ApoE2 (Cys112, Cys158), ApoE3 (Cys112, Arg158), and ApoE4 (Arg112, Arg158), but these seemingly small differences significantly influence ApoE protein structure and function (Mahley, 1988; Weisgraber, 1994) (Figure 1).

A great deal of effort was undertaken to resolve the three-dimensional structure of ApoE protein; however, it was not until 2011 when Chen et al. (2011) unveiled the first human ApoE3 full-length structure, accomplished by nuclear magnetic resonance (NMR) and a series of mutations (R215C/R217S/F257A/W264R/V269A/L279Q/V287E) in order to stabilize ApoE3 in a monomeric form. In summary, ApoE3 forms a helix-bundle structure. The N-terminal domain contains a four-helix bundle. The hinge region, which modulates the interaction of N-terminal and C-terminal domains, contains two helices. The C-terminal domain contains three helices and has a largely exposed hydrophobic surface that is speculated to play a role in initiating the association with lipids and β-amyloid (Aβ) peptides (Chen et al., 2011). Moreover, the X-ray crystal structures revealed that there are only subtle differences in the N-terminal domain of ApoE2, ApoE3, and ApoE4; however, these differences impact the formation of the salt bridge between domains, resulting in their receptor binding variation (Dong et al., 1994; Wilson et al., 1994).

### 2.1 ApoE isoforms in lipid metabolism

ApoE3 and ApoE4 have been shown to exhibit a similar binding affinity for LDLR, which is 50–100 times higher than that of ApoE2 (Weisgraber et al., 1982). The extremely weak interaction of ApoE2 with LDLR is attributed to the amino acid substitution at position 158 from arginine to cysteine. It is postulated that a salt bridge formed between Arg150 and Asp154, which replaces the salt bridge between Arg158 and Asp154, alters the conformation of Arg150, and, as a result, masks the receptor-binding region (residues 136–150) (Wilson et al., 1994). Moreover, the domain interaction between the N-terminal and C-terminal domains is hypothesized to contribute to the differences in lipoprotein-binding preferences among ApoE isoforms. Arginine at position 112 promotes ApoE4 preferentially binding to VLDLs and LDLs. By contrast, the substitution with cysteine at position 112 in ApoE3 and ApoE2 confers an increased specificity for HDLs (Weisgraber, 1990). In ApoE4, a close domain interaction may exist between the N- and C-terminal domains due to a putative salt bridge formed by Arg61 and Glu255. However, this domain interaction may not occur in ApoE2 and ApoE3 due to the existence of Cys112 that buries Arg61 by helices 2 and 3, resulting in a lost connection with Glu255 (Dong et al., 1994; Dong and Weisgraber, 1996) (Figure 1).

Early studies suggested that ApoE2 may promote the development of type III hyperlipoproteinemia by accumulating LDLs in circulation since it defectively binds to LDLR (Horie et al., 1992). It was reported that this increased risk may only happen in 5–10% of individuals carrying two alleles of ApoE2, and the majority of ApoE2 homozygotes were found to be either normolipidemic or hypolipidemic (Mahley et al., 1999). It is speculated that ApoE2 may interact with other genes or environmental factors to drive hypolipidemia to hyperlipidemia. For instance, apolipoprotein A5 S19W mutation was shown to serve as a cofactor in the development of hyperlipidemia in ApoE2 homozygotes (Schaefer et al., 2004). Of particular note, although ApoE4 and ApoE3 have a similar binding affinity for LDLR, when compared with ApoE3, ApoE4 upregulates plasma VLDL levels and increases the risk of atherosclerosis (Knouff et al., 1999). It is thought that the biased binding of ApoE4 for VLDLs may impair the VLDL lipolytic process, causing the decreased VLDL clearance rate in plasma and hence producing a more proatherogenic lipoprotein profile (Li et al., 2013). Overall, the current literature on the role of ApoE isoforms on lipid metabolism has primarily focused on the metabolism of lipoproteins. With emerging revelations about the role of individual lipids, for example, ceramides have been demonstrated to have much better predictive validity than the traditional LDLs and cholesterol measurements for predicting the risk for later occurrence of cardiovascular disease, future research needs to address how ApoE isoforms may impact the metabolism of specific classes of lipids.

### 2.2 ApoE isoforms in AD

A major breakthrough was made in the AD research field in 1993 when Allen D. Roses and colleagues first reported the strong genetic association of *APOE* ε4 with late-onset AD (LOAD), the most common form of the disease (representing 90–95% of AD cases) that does not involve a genetic cause and typically occurs in patients older than 65 years of age (Corder et al., 1993; Saunders et al., 1993; Strittmatter et al., 1993a; Roses, 2006). Since then, the markedly distinct roles of *APOE* genotypes in AD have been substantiated by numerous studies, with ε4 presently being recognized as the greatest genetic risk factor for sporadic AD. Compared with people having 2 copies of ε3, having 1 copy of ε4 triples the AD risk, and the risk is significantly increased to nearly 12-fold with 2 copies of ε4, whereas carrying ε2 reduces the AD risk (Rebeck et al., 1993; Reiman et al., 2020). The mechanisms by which ApoE isoforms differently influence the development of AD have been related to their different roles in the accumulation of toxic Aβ species, the formation of amyloid plaques, the phosphorylation of tau, neuroinflammatory response, and brain utilization of glucose (Langbaum et al., 2009; Koutsodendris et al., 2022). Specifically, brain Aβ levels, amyloid plaques, innate immune neurotoxicity, and tauopathy have been shown to be ApoE isoform-dependent (ε4 > ε3 > ε2) (Maezawa et al., 2006; Bales et al., 2009; Reiman et al., 2009; Shi et al., 2017). Moreover, recent studies from our group demonstrate that ApoE isoforms differentially impact neuronal glycolytic metabolism. Specifically, hexokinase (HK), the gateway enzyme that catalyzes the initial and rate-limiting step of glycolysis, is significantly upregulated by ApoE2, whereas ApoE4 downregulates it. The opposing effects of ApoE2 and ApoE4 on HK drive the disparities in glycolytic activity and general health in neuronal aging, suggesting a potential neuroprotective role for ApoE2 that may arise through the upregulation of neuronal HK expression and glycolytic metabolism (Wu et al., 2018; Zhang et al., 2023).

It is noteworthy that although the *APOE* ε4 allele imposes a significant risk for LOAD, it is not a determinant factor (Corder et al., 1993; Raulin et al., 2022). Over 60% of AD patients carry at least one *APOE* ε4 allele; however, some *APOE4* homozygotes never develop the disease, and some AD patients are *APOE4* negative (Corder et al., 1993; Farrer et al., 1997). It is also worth noting that the association between *APOE* and AD risk varies in different races. Nigerian people have the highest frequency of the *APOE4* allele, which, however, is not found to be associated with an increased risk for AD, likely due to their low cholesterol levels (Sepehrnia et al., 1989; Notkola et al., 1998; Kivipelto et al., 2002; Gureje et al., 2006; Walters et al., 2023; Zhang et al., 2023). Moreover, sex has been shown to modify the risk effect of *APOE* genotypes in AD (Poirier et al., 1993; Farrer et al., 1997). Amounting evidence indicates that females with *APOE4* have a greater risk of developing AD than male *APOE4* carriers (Beydoun et al., 2012; Liew, 2022). It may be in part due to a higher *APOE* ε4 association with the expression of total and phosphorylated tau in females than in males (Hohman et al., 2018). In addition, loss of female sex hormones during menopause constitutes an additional susceptibility factor that can potentially heighten the negative impact of *APOE* ε4 on female brains (Zhao et al., 2016; Chhibber and Zhao, 2017; Mosconi et al., 2017; Zhang et al., 2021). In regards to the sex effect on *APOE* ε2, although Altmann et al. (2014) found a protective role of *APOE2* in male but not in female subjects, a recent study reported a more pronounced neuroprotective effect of *APOE2* in females than in males (Lamonja-Vicente et al., 2021). The latest study, which involved 32,427 participants, including 59% females, 14% non-Hispanic black individuals, and 86% non-Hispanic white individuals, revealed that *APOE* ε4-related increased AD risk in females is not altered by race; however, *APOE* ε2-mediated protective effect appeared to be more evident in female white individuals and male black individuals (Walters et al., 2023). In summary, despite the well-established significant roles of *APOE* genotypes in AD and elucidation of numerous processes that can be affected by ApoE isoforms, recent studies have highlighted the roles of other players, including sex, race, and environmental factors that can amplify or cancel the *APOE* effects on AD. Uncovering other key players and associated pathways will undoubtedly lead to insights into new avenues for AD intervention.

## 3 ApoE rare variants

### 3.1 Anti-AD ApoE variants

In recent years, several rare variants of ApoE have received great attention for their unique associations with AD. One such variant is the Christchurch variant, in which the residue at position 136 is substituted from arginine to serine. The *APOE*-Christchurch (*APOE*Ch) variant was first discovered in 1987 in an *APOE2* patient with type III hyperlipoproteinemia. This patient was treated in the Christchurch Lipid Clinic, and thus, the variant was initially named *APOE2*Ch (Wardell et al., 1987). The patient was heterozygous for *APOE2*Ch (R136S), which is associated with VLDLs at a level approximately five-fold higher than the wildtype *APOE2*, suggesting that the *APOE2*Ch variant might have contributed to the patient’s hyperlipoproteinemic condition (Wardell et al., 1987). Subsequently, *APOE2*Ch variant-related studies were mostly focused on lipid metabolism, which overall indicated that *APOE2*Ch variant was not necessarily connected with lipid metabolism disorders (Pocovi et al., 1996; Hubácek et al., 2000, 2002, 2005). Strikingly, in 2019, a unique case report documented a Colombian woman who carried a homozygous *APOE3*Ch variant and survived with a three-decade delay in the onset of AD dementia that would have been caused by her *PSEN1* E280A mutation (Arboleda-Velasquez et al., 2019). Postmortem analyses revealed that her brain had a high amyloid load but with limited tau pathology and neurodegeneration (Arboleda-Velasquez et al., 2019). The residue altered in the *APOE*Ch variant, Arg136Ser, is localized within the receptor binding region (residue 136–150), which is also where ApoE interacts with HSPG (residues 142–147) (Weisgraber et al., 1986; Mahley and Ji, 1999; Mahley et al., 1999; Saito et al., 2003; Arboleda-Velasquez et al., 2019). HSPG has been implicated in AD pathogenesis (Leveugle et al., 1998; Liu et al., 2016), and ApoE4 as the major risk of AD has a strong binding with HSPG (Futamura et al., 2005). *APOE3*Ch variant exhibited an impaired heparin binding compared to other *APOE* isoforms, suggesting that reduced interaction with HSPG may have contributed to the mechanism of the AD protective effect of *APOE3*Ch variant found in the Colombian woman (Arboleda-Velasquez et al., 2019; Bu, 2022). In support of this mechanism, Marino et al. (2023) recently developed and characterized an antibody, 7C11, which inhibited the interaction between ApoE4 and HSPG, and effectively attenuated ApoE4-induced cytotoxicity and tau phosphorylation.

In 2014, Medway et al. (2014) reported the AD protective variant, *APOE3*-V236E, in which the valine residue at position 236 of *APOE3* is substituted with glutamate. Interestingly, the V236E mutation was initially associated with ApoE2 and none of the carriers of *APOE2*-V236E exhibited lipoprotein profile abnormalities (van den Maagdenberg et al., 1993; Zhao et al., 1994). ApoE at 236 position is not only close to the lipid-binding region (244–272) and it is localized within a region (230–243) that is vital for ApoE oligomerization and Aβ binding (Frieden and Garai, 2012), suggesting that the AD protective effect of *APOE3*-V236E mutation may be achieved through regulation of lipid metabolism, ApoE aggregation, and Aβ clearance. Liu et al. (2021) further demonstrated the mechanism of *APOE3*-V236E mutation against AD included downregulation of ApoE4 self-aggregation, enhancement of ApoE lipidation, and decreased amyloid deposition toxicity, which together promoted healthy brain aging. *APOE3*-V236E mutation was named as *APOE3*-Jacksonville variant based on the city where the study was primarily conducted.

In 2022, Le Guen et al. (2022) reported a new AD protective variant, *APOE4*-R251G, in which the mutation occurs in the lipid-binding region (244–272); the arginine residue at position 251 of *APOE4* is replaced with glycine, and this mutation has been shown to mitigate the ε4-associated AD risk. *APOE4*-R251G mutation was first reported during a hyperlipidemia screening; carrying this mutation was linked to lipid dysmetabolism (van den Maagdenberg et al., 1993). Moreover, *APOE4*-R251G mutation was found in a family with hyperlipidemia and coronary heart disease; elevated LDL and upregulated ratio of VLDL cholesterol to total triglycerides were found in carriers of this variant (Kang et al., 1997). Considering the location of R251G in the lipid-binding region of ApoE4, it can be speculated that the protective mechanism of *APOE4*-R251G in AD may be related to favorable changes in brain lipid metabolism that increases brain resistance to AD compared to wild type ApoE4.

### 3.2 Pro-AD ApoE variants

Apart from the above described ApoE variants that are found to be protective against AD, other variants of ApoE that may increase the AD risk have also been reported. In 1999, a rare ApoE4 mutant, *APOE4*-L28P Pittsburgh variant, in which the substitution from leucine to proline occurs at position 28 of *APOE4*, was discovered in the heterozygous form when screening the ApoE mutants in white LOAD patients who were from Pittsburgh, Indiana, and Mayo Clinic Rochester (Kamboh et al., 1999). Researchers found that carrying the *APOE4*-L28P Pittsburgh mutant increased the AD risk to five-fold higher than carrying wild type *APOE4* (Kamboh et al., 1999). Moreover, a study conducted in Italy that screened *APOE* mutation in LOAD patients also observed that carrying the *APOE4*-L28P mutation had a higher risk of developing AD (Scacchi et al., 2003). The mechanistic study further revealed that the *APOE4*-L28P Pittsburgh variant significantly changed the structure, conformation, and function of ApoE4 (Argyri et al., 2014). However, the association between *APOE4*-L28P mutation and AD risk was not found in the Spanish population (Baron et al., 2003) and European Americans (Medway et al., 2014).

More recently, Le Guen et al. (2023) reported a new ApoE3 variant, *APOE*3-R145C, which harbors a mutation from arginine to cysteine at position 145 of ApoE3 and was shown to increase the risk of AD among African-ancestry individuals with the ε3/ε4 genotype. Over 4% of African ancestry populations carry the R145C mutation, while European ancestry rarely possesses this mutation (Abou Ziki et al., 2014). *APOE*3-R145C/*APOE4* carriers have a similar level of AD risk with *APOE* ε4/ε4 carriers among the African-ancestry populations; however, no increased AD risk was observed among individuals with *APOE* ε3/ε3 having the R145C mutation, suggesting that the *APOE3*-R145C mutation may alter certain function of ε3 that heightens the vulnerability of a brain that already has a predisposed high risk conferred by ApoE4 for AD (Le Guen et al., 2023). The R145C mutation also exists in *APOE4* and *APOE2*, which are closely associated with type III hyperlipoproteinemia (Lohse et al., 1992; de Villiers et al., 1997). Furthermore, it was reported that the R145C mutation decreases ApoE binding with LDLR and VLDL receptors, which are vital for lipid metabolism (Rall et al., 1982a; Ruiz et al., 2005). Thus, it hints that the higher AD risk in *APOE*3-R145C/*APOE4* carriers among African-ancestry individuals may also be related to dysregulated lipid metabolism.

In summary, efforts in identifying and elucidating the roles of rare variants of ApoE in promoting brain resilience or brain susceptibility for AD have yielded novel insights into understanding the mechanisms underlying the distinct impact of ApoE isoforms in AD pathogenesis and devising potential strategies to mitigate ApoE4-mediated risk. However, the current findings remain preliminary due to the minimal number of clinical cases analyzed and thus need to be further validated in larger study cohorts. Moreover, how these variants impact the ApoE structure and its function, whether the variants have gender or race specific effects, and how they influence the progression of AD, are all important questions that warrant further investigations.

## 4 ApoE protein glycosylation and sialylation

Glycosylation attaches carbohydrate moieties enzymatically, and it is the most complex PTM of protein, adding tremendous proteomic diversity and functionality. The complexity of protein glycosylation is first reflected in the different types of glycosylation with distinct glycosidic linkages. The second layer of complexity reflects on the molecular events and various enzymes involved in each glycosylation type. For example, a typical *N*-linked glycosylation process consists of four separate events: precursor glycan assembly, glycan attachment, glycan trimming, and glycan maturation; each represents multiple enzymatic steps. In comparison, *O*-linked glycosylation targets different residues, and the glycosylation method involves sugars that are being added one at a time. The complexity of glycosylation is further expanded by the highly diverse glycan structures with variable number, composition, and arrangement of sugar molecules in each specific glycan chain. Moreover, proteins are often glycosylated at multiple sites with different glycosidic linkages and diverse glycan structures, and a given protein can exhibit distinct glycosylation patterns in a species, tissue, and cell-specific manner, partly due to enzyme availability and specificity. As a result of this vast complexity and heterogeneity, detecting and characterizing protein glycosylation has been an extremely challenging task until recent years, with significant progress made with the development of more advanced and sophisticated methodology (Stanley, 2011; Schedin-Weiss et al., 2014; Varki, 2016; Reily et al., 2019).

Extracellular proteins are often modified with *N*-glycans on asparagine (Asn or N) residue and *O*-glycans on serine/threonine (Ser/Thr or S/T) residues (Wiederschain, 2009). A consensus motif, Asn-*X*-Ser/Thr, has been established for *N*-glycosylation; however, an apparent consensus motif for *O*-glycosylation does not seem to exist. Protein glycosylation plays an essential role in various biological processes, including protein folding, secretion, uptake, intracellular sorting, cell–cell interaction, and host-microbial recognition (Ohtsubo and Marth, 2006; Grewal et al., 2008). *N*- and *O*-glycans are frequently terminated with sialic acids, with *N*-acetyl-5-neuraminic acid (Neu5Ac) being the dominant form in human glycoproteins. Sialic acids are unique in that they are negatively charged carbohydrates and have been shown to serve as a critical mediator of molecular recognition and interaction (Keppler et al., 1999; Yoo et al., 2015). ApoE protein is known to be highly glycosylated, and carbohydrate moieties are attached exclusively through *O*-linkage on Ser/Thr residues. Historically, ApoE glycosylation has been largely neglected until recent years, and important progress has been made in understanding its glycosylation sites, glycan structure, and biological function.

### 4.1 Identification of *O*-glycosylation sites in human ApoE

In 1980, Zannis and Breslow (1980) described a complex array of ApoE isoproteins associated with VLDL, which differed from each other in molecular size, isoelectric point, or both, on a high-resolution, two-dimensional gel electrophoresis system. In the following study, the same authors reported that the previously observed ApoE isoprotein polymorphism arose in part from the PTM, such as sialylation of the protein, in addition to the contribution of the genetic variability of ApoE (Zannis and Breslow, 1981). These earlier studies paved the path for the ultimate identification of the first glycosylation site at Thr194 in human ApoE (Wernette-Hammond et al., 1989). Over the last three decades, 10 additional studies were conducted, which, together with the initial report, led to the discovery of eight glycosylation sites in human ApoE (Table 1).

**Table 1:** Identification of posttranslational glycosylation sites on human ApoE.

Study (author, year)	Glycosylation residues identified in the study	Sample source of human ApoE	Detection methods	Major findings
Wernette-Hammond et al. (1989)	Thr194	Plasma ApoE was obtained from a type V hyperlipoproteinemic subject with an ApoE3/3 genotype ApoE3 and ApoE3 mutant (Thr194 → Ala194) were produced in HeLa and CHO cells by transient transfection	1-D gel electrophoresis (1-DE) 2-D gel electrophoresis (2-DE) Amino sugar and sequence analyses Site-directed mutagenesis (SDM)	Thr194 in plasma ApoE is *O*-glycosylated Thr194 in plasma ApoE exists in three sialoforms: asialo (non-glycosylated), monosialo, and disialo Glycosylation of Thr194 is not essential for ApoE secretion
Mancone et al. (2007) ^a^	Thr194	Plasma VLDL was obtained from three healthy donors with an ApoE3/3 genotype	2-DE MALDI-TOF/TOF	Plasma VLDL contains at least five different electrophoretic variants of ApoE Thr194 in VLDL ApoE exists in at least three *O*-glycoforms: non-glycosylated, glycosylated by a HexNAc-Hex-NeuAc structure, or a HexNAc-Hex-(NeuAc)_2_ structure
Atkinson et al. (2009)	Thr194	Human serum was obtained from subjects with an ApoE3/3 genotype: *n* = 12 (with preeclampsia at 36–38 weeks) *n* = 12 (age-matched healthy control participants)	2-DE LTQ FT-ICR	Compared to control serum, ApoE with Thr194 *O*-glycosylated with HexNAc-Hex-NeuAc is downregulated, whereas non-glycosylated-ApoE is upregulated in preeclampsia serum
Nilsson et al. (2009)	Thr194 Thr289, Ser290	Human CSF (*n* = 3)	Sialic acid capture and release Reversed-phase liquid chromatography (RPLC)/ESI-FTICR	The sialic acid capture-and-release strategy enriches and increases the purity of glycopeptides, which facilitates MS detection of novel sites of glycosylation and core glycan structures on sialylated glycoproteins In addition to the previously identified Thr194, two new *O*-glycosylation sites at Thr289 and Ser290 are found on human CSF ApoE; all three residues are modified by sialylated core 1 glycan structures
Lee et al. (2010)	Thr194 Ser290 Thr289/Ser296^b^	Human monocyte-derived macrophages were obtained from donors with an ApoE3/3 genotype	Immunoprecipitation 1-DE and 2-DE Lectin blot analysis Neuraminidase treatment Nano-lC/LTQ-FT Ultra	Thr194 is identified to exist in eight *O-*glycoforms and contains (HexNAc)_2_-Hex_2_-(NeuAc)_2_ as the most complex glycan structure The C-terminus *O*-glycosylation residues (Ser290 and putative sites at Thr289 and Ser296) are relatively less extensively modified with less complex glycan structures than Thr194 Macrophage-derived ApoE is more sialylated than plasma ApoE The maximum number of sialic acid residues detected in individual glycans is 2
Steentoft et al. (2011) ^a^	Thr194 Thr289, Ser290, Ser296	Human T3M4 cells	Zinc-finger nuclease (ZFN) gene targeting lectin weak affinity chromatography (LWAC) Neuraminidase treatment Nano-LC-ESI-MS/MS	ZFN targeting of COSMC produces truncated HexNAc or HexNAc-NeuAc *O*-glycans, which, in combination with LWAC isolation and neuraminidase treatment, increases MS detection coverage of HexNAc-type *O-*glycopeptides Human ApoE is *O*-glycosylated by core 1 HexNAc structures at Thr194, Thr289, Ser290, and Ser296
Halim et al. (2013) ^a^	Thr8, Thr18 Thr194 Thr289, Ser290, Ser296	Human CSF (*n* = 8)	PNGase F pretreatment Sialic acid capture and release Nano-LC-ESI-MS/MS	PNGase pretreatment enriches and facilitates MS analyses of *O*-glycoproteins Human CSF ApoE contains six sialylated *O*-glycosylation sites, including two minor sites at Thr8 and Thr18 These residues are predominantly modified by core-1-like HexHexNAc structures Thr194 contains core-2-like Hex(HexHexNAc)HexNAc structures 108 *O*-glycosylation sites are identified from CSF proteins, which occur predominantly on Ser/Thr residues that have Pro residues at positions: P-S/T, S/T-P, or S/T-X-X-P
Uen et al. (2015)	Ser129	Plasma was obtained from Taiwanese breast cancer patients (*n* = 24; average age 46 years) and age-matched female control subjects (*n* = 24; average age 57 years)	1-DE Nano-HPLC/LTQ-Orbitrap	Ser129 in plasma ApoE is found to be slightly more glycosylated (1.14-fold) in Taiwanese breast cancer patients when compared to age-matched female control subjects
Zhu et al. (2019)	Ser76/Thr83/Thr89^b^ Ser129/Thr130^b^ Thr194, Ser197 Ser263 Thr289/Ser290^b^ Ser296	Plasma HDL was obtained from healthy human subjects (*n* = 10; 5 females, 5 males; 18–25 years old)	HPLC_QQQ MRM	Plasma HDL ApoE is heavily *O*-glycosylated at seven sites: Thr194, Ser197, Ser263, Ser296, and three putative sites at Ser76/Thr83/Thr89, Ser129/Thr130, and Thr289/Ser290 Within the hinge region, Ser197 is far more extensively glycosylated than Thr194 Thr194 contains only one HexNAc structure; in stark contrast, 23 distinct glycan structures are detected on Ser197, including previously unidentified fucosylated structures
Flowers et al. (2020)	Thr8, Thr18 Thr194, Ser197 Thr289, Ser290, Ser296	CSF ApoE (*n* = 4; mean age 58 years) and plasma ApoE (*n* = 4; mean age 67 years) were obtained from healthy subjects	Immunoprecipitation LC/QTOPF-MS/MS	Both CSF and plasma ApoE are *O*-glycosylated at seven sites Thr8 at the N-terminus is more abundantly glycosylated by monosialylated core 1 glycans in plasma ApoE than in CSF ApoE Residues (Thr289, Ser290, Ser296) at the C-terminus are more abundantly glycosylated by monosialylated and disialylated core 1 glycans in CSF ApoE than in plasma ApoE Residues (Thr194, Ser197) within the hinge region are modified by three core 1 glycans: unsialylated HexNAc-Hex, monosialylated HexNAc-Hex-NeuAc, and disialylated HexNAc-Hex-(NeuAc)_2_, and the modification exhibits similar abundance in CSF and plasma ApoE
Ke et al. (2020)	Ser94 Thr194 Thr289	Plasma LDL L1 (least negatively charged and no harmful effects) and L5 (most negatively charged and pro-atherogenic) subfractions were obtained from human subjects with metabolic syndrome (*n* = 6) and healthy controls (*n* = 3)	2-DE Nano UPLC/MS	ApoE in L1 versus L5 subfractions of plasma LDL exhibits distinct glycosylation patterns ApoE in L5 subfraction is *O*-glycosylated at Ser94, Thr194, and Ser289; all contain a HexNAc-Hex-NeuAc glycan sequence. In stark contrast, no glycosylation is detected in ApoE in L1 subfraction

Representation of glycan residues: HexNAc, *N*-acetylhexosamine [*N*-acetylgalactosamine (GalNAc) or N-acetylglucosamine (GlcNAc)]; Hex, galactose or Gal; NeuAc, *N*-acetylneuraminic acid or sialic acid.

a Glycosylation residues are numbered based on the full-length ApoE preprotein (317-amino-acid length including the signal peptide).

b Putative glycosylation sites.

### 4.2 Hinge region

As described above, ApoE protein is composed of two discrete structural domains connected by a hinge region (residues 168–205): N-terminal domain (residues 1–167) including a highly positively charged receptor-binding region, and C-terminal domain (residues 206–299) containing a lipid-binding region (Wilson et al., 1991; Sivashanmugam and Wang, 2009; Chen et al., 2011). Studies have noted that the hinge region in ApoE is a relatively unstructured and flexible segment, and it is highly susceptible to proteolysis (Wetterau et al., 1988). Two *O*-glycosylation sites (Thr194 and Ser197) within the hinge region in ApoE have been identified. The glycosylation and sialylation status of these residues can potentially have a significant impact on the physiochemical properties and, consequently, biological activities of the hinge region. For example, the interaction between ApoE and TREM2, another major risk factor for sporadic AD, has been reported to facilitate the uptake of Aβ by microglia, and AD-linked mutations in TREM2 impair the binding affinity of TREM2 for ApoE leading to Aβ accumulation in the brain (Yeh et al., 2016). The molecular recognition of the interaction between ApoE and TREM2 possibly involves the hinge region in the ApoE protein, particularly residues 192–238, in an isoform-dependent manner (Kober et al., 2020; Mai et al., 2022). In a larger context, the conformational dynamics of N- and C-terminal domains conferred by the hinge region flexibility can likely influence the general function of ApoE (Morrow et al., 2000; Frieden et al., 2017).

Thr194 was the first *O*-glycosylation site identified in human plasma ApoE3 using several complementary approaches, including both one- and two-dimensional gel electrophoresis, amino sugar and sequence analyses, and site-directed mutagenesis. Specifically, Thr194 in ApoE3 was characterized to be *O*-glycosylated and existed in three major isoforms: asialo also non-glycosylated isoform, monosialo isoform containing a relatively simple carbohydrate structure, and disialo isoform containing a more complex and branched carbohydrate structure. Moreover, the glycosylation of Thr194 was shown to be not essential for ApoE3 secretion (Wernette-Hammond et al., 1989). The Thr194 residue as a major *O*-glycosylation site in human ApoE was repeatedly confirmed in many of the later studies. The second study published nearly 20 years later represented the first proteomics work that attempted to comprehensively unravel the PTM of human ApoE associated with lipoproteins. Two-dimensional gel electrophoresis followed by MALDI-TOF/TOF analyses detected five ApoE isoforms in VLDL isolated from the plasma of ApoE ε3/ε3 donors. Three isoforms were identified to contain Thr194 with no glycans (isoform 1) or attached with distinct glycan structures, HexNAc-Hex-NeuAc (isoform 2), or HexNAc-Hex-(NeuAc)_2_ (isoform 3) (Mancone et al., 2007). More recent works conducted in primary human cell cultures, serum, and CSF have also been able to consistently define that the *O*-glycans attached to Thr194 are terminated with the maximum number of sialic acid residues per glycan (Sugano et al., 2008; Atkinson et al., 2009; Nilsson et al., 2009; Lee et al., 2010; Steentoft et al., 2011; Halim et al., 2013; Ke et al., 2020). In particular, in human monocyte-derived macrophages, Thr194 was found to exit in eight different glycoforms containing the complex glycan structures such as (HexNAc)_2_-Hex_2_-(NeuAc)_2_ (Lee et al., 2010). The specific function of glycosylation of Thr194 in ApoE is unknown. A recent structural prediction study indicated that Thr194 is hydrophobic and functions as a linker between the LDLR binding site and C-terminal lipid-binding region, raising the possibility of the influence of the PTM status of Thr194 in ApoE-mediated lipid metabolism (Ke et al., 2020).

Ser197 has recently been identified as the second *O*-glycosylation site in the hinge region of ApoE (Zhu et al., 2019; Flowers et al., 2020). Of particular note, in the 2019 study reported by Zhu et al. (2019), among the seven glycosylation sites detected in HDL-associated ApoE, Ser197 was found to be the most extensively glycosylated residue. It becomes even more striking when Ser197 is compared to Thr194, a residue that had previously been established as the major glycosylation site in human ApoE. In the Zhu et al. (2019) study, Thr194 was found to be minimally glycosylated with only one GalNAc structure detected; in stark contrast, a total of 23 distinct glycan structures were detected on Ser197, from simple GlcNAc to biantennary structures containing both sialic acid and fucose. In the 2020 study reported by Flowers et al. (2020), Thr194 and Ser197 were found to be both glycosylated in CSF and plasma ApoE. Although Thr194 was characterized to be more heavily glycosylated than Ser197, these two residues are similarly modified by three core 1 glycan structures (nonsialylated, monosialylated, and disialylated), and the relative proportions of these glycans were not significantly different between the CSF and plasma ApoE. Overall, due to the structural flexibility of the hinge region, Thr194 and Ser197 may be more readily exposed with better accessibility to substrates and enzymes for glycosylation and sialylation, making them two major *O*-glycosites in human ApoE (Hatters et al., 2006; Mahley et al., 2009; Frieden et al., 2017). The reported disparities in their abundance in glycosylation may indicate their tissue specificity and binding preference; for example, it is possible that glycosylation of Ser197 is particularly important for ApoE association with HDL particles.

### 4.3 N-terminal domain

The first 18 amino acids at the N-terminus of human ApoE protein serve as the signal peptide sequence that functions to direct newly synthesized ApoE to the Golgi apparatus for PTMs such as glycosylation and sialylation, and it gets cleaved before secretion of mature ApoE (Rall et al., 1982b; McLean et al., 1984; Zannis et al., 1984). The identification of glycosylation sites in the N-terminal domain of ApoE has proved to be challenging, which may be in part due to the interference from the N-terminal processing in the secretory pathway. Alternatively, the presence of *O*-glycans in the vicinity of Lys/Arg residues may block the access of trypsin to cleave the peptide bond, causing the corresponding glycopeptides to miss out in the following MS analysis (Halim et al., 2013).

Halim et al. (2013) first identified two minor *O*-glycosylation sites at Thr8 and Thr18 of the N-terminal domain of ApoE in human CSF. This elegantly executed study is unique in that it first carried out a pretreatment of CSF proteins with peptide *N*-glycosidase F (PNGase F), which removed *N*-glycans from glycoproteins, allowing selective characterization of *O*-glycopeptides. Following the PNGase F pretreatment, a sialic acid capture-and-release protocol was applied, which enriched sialylated *O*-glycopeptides for the subsequent MS analysis. CSF ApoE was identified to contain six *O*-glycosylation residues, mostly modified by core 1 HexHexNAc glycan structures. Additionally, the study detected a total of 106 *O*-glycosylation sites in CSF proteins, and they predominantly occurred on Ser or Thr residues that had a Pro residue at one of the three positions: P-S/T, S/T-P, and S/T-X-X-P, pointing toward a potential consensus motif for *O*-glycosylation that warrants further investigations (Halim et al., 2013). Flowers et al. (2020) also reported that both Thr8 and Thr18 were *O*-glycosylated by sialylated core 1 HexHexNAc structures in CSF and plasma ApoE, although the site occupancy in plasma ApoE was found to be much higher than in CSF ApoE.

Ser129, located near the receptor-binding region (residues 136–150), was first identified in Taiwanese women, which exhibited a slight increase in glycosylation in the plasma of patients diagnosed with stage II breast cancer compared to age-matched female control subjects (Uen et al., 2015). A putative glycosylation site at Ser129/Thr130 was also associated with HDL ApoE (Zhu et al., 2019). Recent studies have indicated two additional glycosylation sites in the N-terminal domain of ApoE, including Ser94 (Ke et al., 2020), and a putative site at Ser76/Thr83/Thr89 (Zhu et al., 2019). Compared to other glycosylation sites that have been confirmed by at least two independent studies, these two sites were respectively reported in only one study and need to be further characterized in future studies. Functionally, elucidation of the potential impact of the glycosylation and sialylation status of the N-terminal domain on ApoE interactions with ApoE receptors such as LDLR and HSPG may aid understanding of the distinct roles of ApoE isoforms in lipid and tau metabolism.

### 4.4 C-terminal domain

The C-terminal domain (residues 225–299) of ApoE is responsible for lipid binding (Weisgraber, 1994; Mahley and Rall, 2000). ApoE isoforms have been shown to have significantly divergent specificities in their associations with different lipoproteins. ApoE2 and ApoE3 bind preferentially to small phospholipid-rich HDL, while ApoE4 is found to bind preferentially to large triglyceride-rich VLDL (Nguyen et al., 2010). Three *O*-glycosylation residues at the C-terminus of ApoE have been established in multiple studies. In addition, glycosylation of Ser263 was also reported (Zhu et al., 2019). A clear definition of the glycosylation and sialylation characteristics of these residues can help better understand the lipid binding properties of ApoE.

Nilsson et al. (2009) first reported two *O*-glycosylation sites at the C-terminus of human CSF ApoE, Thr289 and Ser290, and they were found to be modified by sialylated core 1 structures. The detection of the two new sites at the C-terminus might have benefited from the sialic acid capture-and-release procedure, which increased the purity of glycopeptides and, as a result, facilitated MS analyses of new sites of glycosylation on sialylated glycoproteins. In an independent study reported by Lee et al. (2010), Ser290 was also identified to be *O*-glycosylated in ApoE expressed in human monocyte-derived macrophages. In addition, the study identified a putative *O*-glycosylation site at the C-terminus of ApoE in human primary macrophages, Thr289/Ser296. All these three C-terminus glycosylation residues (Thr289, Thr290, and Ser296) were subsequently reported in ApoE expressed in human T3M4 cells (Steentoft et al., 2011), CSF ApoE obtained from older individuals (Flowers et al., 2020), and ApoE associated with HDL (Zhu et al., 2019). Compared to Thr289 and Ser290, Ser296 was characterized as a minor glycosylation site in ApoE from human CSF (Flowers et al., 2020).

In glycoprotein modeling, sialylation in the C-terminal region was predicted to alter ApoE’s hydrophobicity and interactions with lipids (Flowers et al., 2020). Specifically, the decreased hydrophobicity and increased electronegativity conferred by Thr289 sialylation could lead to structural changes that impair the ability of ApoE in the regulation of lipid transport and metabolism (Ke et al., 2020). Ser290 is located just after the last C-terminal helix at the beginning of an unstructured C-terminal tail; therefore, sialylation of Ser290 is unlikely to disrupt the interhelical connections. However, the presence of strongly negatively charged sialic acid residues on Ser290 may weaken the interactions between ApoE molecules and consequently prevent ApoE self-association and aggregation (Lee et al., 2010).

### 4.5 ApoE protein glycosylation and sialylation: species variability

**Figure 2:**
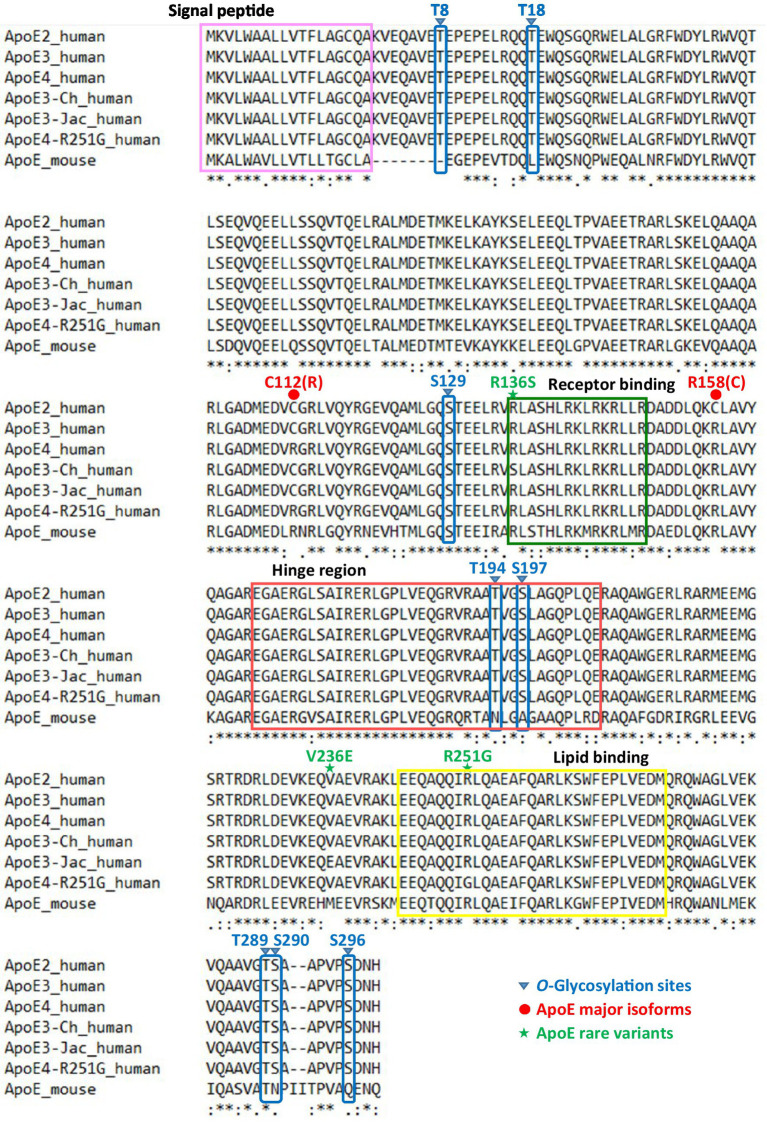
Protein sequence alignment of human ApoE (ApoE2, ApoE3, ApoE4, and AD-related rare variants) and mouse ApoE. The first 18 amino acids serve as the signal peptide that directs newly synthesized ApoE preprotein (317-amino-acid length) to the Golgi apparatus for posttranslational modifications and it is cleaved before secretion of mature ApoE (299-amino-acid length). A total of eight *O*-linked glycosylation sites on human ApoE have been experimentally identified by at least two independent studies: three residues (Thr8, Thr18, Ser129) localized in the N-terminal domain, two residues (Thr194, Ser197) localized within the hinge region, and three residues (Thr289, Ser290, Ser296) localized in the C-terminal domain. Of the eight *O*-glycosites on human ApoE, only two residues (Ser129, Thr289) are conserved in mouse ApoE.

**Figure 3:**
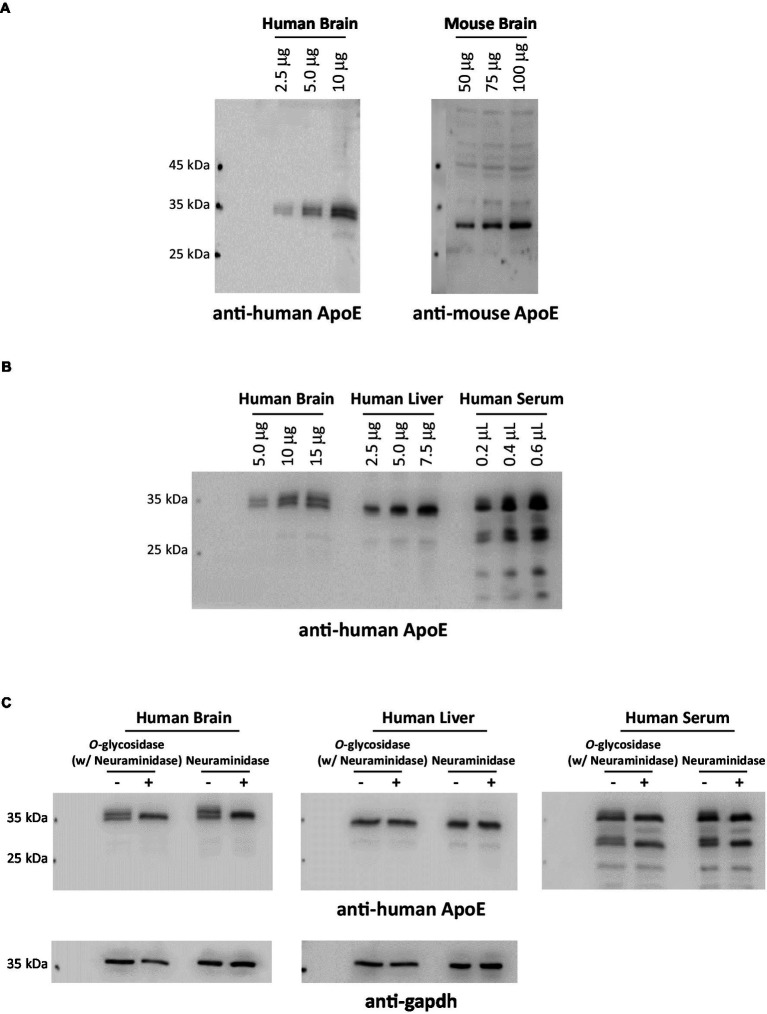
ApoE protein glycosylation and sialylation profiles exhibit distinct variations across species and organs. **(A)** Human (with an ApoE3/3 genotype) and mouse brain cortex lysates were comparatively analyzed by immunoblotting and probed with anti-human ApoE or anti-mouse ApoE antibodies, respectively. The data indicates that ApoE in the human brain is extensively glycosylated and sialylated; in contrast, ApoE in the mouse brain is non-glycosylated or sparingly glycosylated. **(B)** Human postmortem brain cortical lysates, human liver whole tissue lysates, and human serum were comparatively analyzed by immunoblotting and probed with anti-human ApoE. **(C)** Human brain lysates, liver lysates, and serum were deglycosylated or desialylated by endoglycosidases (a combination of *O*-glycosidase and neuraminidase or neuraminidase alone) at 37°C for 1 h. ApoE glycosylation and sialylation were analyzed by immunoblotting. Blots were probed for anti-human ApoE immunoreactivity. The data presented in **(B,C)** indicate that a significantly greater percentage of ApoE in the human brain undergoes glycosylation and sialylation than ApoE in the circulation. Interestingly, ApoE in the human liver is nearly non-glycosylated.

Since the first site was identified in 1989, nearly a dozen *O*-glycosylation residues in human ApoE protein have been experimentally characterized (Table 1). Of them, eight residues (Thr8, Thr18, Ser129, Thr194, Ser197, Thr289, Ser290, and Ser29) have been confirmed by at least two independent studies. The protein sequence alignment shows 71.6% identity between human and mouse ApoE; however, of the eight *O*-glycosylation residues found in human ApoE, only two residues, Ser129 and Thr289, are conserved *O*-glycosylation residues also present in mouse ApoE (Figure 2). Consistent with the variability in the *O*-glycosylation residues available in human versus mouse ApoE, Western blot analyses revealed significant differences in ApoE expression patterns in human versus mouse cortex lysates. Multiple immunoreactive bands of ApoE protein were detected in the human cortex lysates, indicating the ApoE protein in the human brain is highly glycosylated and sialylated. In contrast, only one band at a lower molecular weight was observed in the mouse cortex lysates, indicating ApoE in the mouse brain is minimally glycosylated and sialylated (Figure 3A). Hussain et al. (1988) reported similar observations that despite extensive glycosylation of ApoE in CSF and human brain tissue, murine brain ApoE was sparingly glycosylated. This variability exhibited in human versus mouse ApoE underscores the importance of research models in studies of glycobiology and sialobiology of human ApoE.

### 4.6 Human ApoE protein glycosylation and sialylation: tissue variability

Further evidence indicates tissue variability of glycosylation and sialylation in human ApoE. To elucidate central versus peripheral differences, we conducted a comparative analysis of ApoE expression profiles in human brain lysates, whole liver lysates, and serum, and the data indicate that ApoE expression patterns in these samples are significantly disparate. Multiple ApoE immunoreactive bands were detected in brain lysates and serum; in stark contrast, only one major band was observed in liver lysates. Additionally, ApoE from brain lysates showed more distinct upper bands than ApoE from human serum (Figure 3B). To verify the upper bands are sialylated forms of ApoE, all three samples were treated with neuraminidase with and without *O*-glycosidase. As expected, neuraminidase treatment resulted in the disappearance of the upper bands of ApoE in human brain lysates and serum; however, no change was detected in human liver lysates (Figure 3C). These data indicate that ApoE protein in the human brain and blood contain sialylated ApoE; in contrast, ApoE protein in the human liver appears to lack sialylation. These findings are intriguing. In the periphery, ApoE protein is mainly synthesized and secreted by the liver, contributing to approximately 75% of peripheral pools of ApoE (Huang and Mahley, 2014). Moreover, it is well-documented that plasma ApoE is largely liver-derived (Williams et al., 1985; Mahley, 1988). Then, what may have accounted for the differences in the glycosylation and sialylation patterns of ApoE in human serum and liver lysate? It can be speculated that ApoE in the circulation could come from multiple sources; in addition to the liver, monocytes, and macrophages could be other major sources of ApoE in the blood (Hatters et al., 2006; Hauser et al., 2011), giving rise to a mixture of sialylated and non-sialylated ApoE in human serum. Further research is necessary to explore how human ApoE glycosylation and sialylation occur differently in a tissue- and cell-specific manner.

### 4.7 Human ApoE protein glycosylation and sialylation: isoform variability

Recent studies indicate that human ApoE glycosylation and sialylation can also vary among isoforms, which can consequently impact the biological function of ApoE isoforms. Flowers et al. (2020) conducted a comprehensive analysis of the proportion of ApoE glycosylation between the CSF and plasma in a cohort of healthy individuals. The glycosylation proportion of ApoE was found to be markedly different between the CSF and plasma, depending on the locations of the glycosylation residues in the ApoE protein. Interestingly, plasma ApoE exhibited much more extensive glycosylation (20.5%) compared to CSF ApoE (0.1%) at the N-terminus of ApoE. In stark contrast, CSF ApoE held nearly 10 times more glycosylation (36.8%) than plasma ApoE (3.8%) at the C-terminus of ApoE. In the hinge region, 27.3% of glycosylated peptides were detected in CSF ApoE compared to 10.3% in plasma ApoE (Flowers et al., 2020). Consistent with the Flowers report, Lawler et al. (2023) showed that the *O*-glycosylation site occupancy in CSF ApoE was 10.6% compared to 4.3% in plasma ApoE in the hinge region. Similarly, CSF ApoE carried four times more glycosylation (22%) than plasma ApoE (5.4%) in the C-terminal domain of ApoE. Additional evidence of CSF ApoE undergoing much more extensive glycosylation than plasma ApoE was presented by the study conducted by Hu et al. (2020), which found that approximately 66.5% of ApoE in CSF were glycosylated forms compared to only 12.5% of ApoE in plasma. Moreover, it was found that the highest percentage of glycosylated forms was associated with ApoE2 (13.7%), followed by ApoE3 (12.7%) and ApoE4 (9.96%) in plasma, and the same trend was observed in CSF with the order of ApoE2 (69.7%) > ApoE3 (67.2%) > ApoE4 (65.7%) (Hu et al., 2020). The most recent study further confirmed that ApoE glycosylation profiles are isoform-specific, with a lower extent of ApoE4 glycosylation in CSF but not in plasma (Meuret et al., 2023).

Several studies have attempted to distinguish ApoE sialylation profiles rather than total glycosylation. Conejero-Goldberg et al. (2014) reported that ApoE protein levels in human tissues varied among ApoE isoforms, with ApoE2 being the most abundant, though mRNA expression levels did not alter, suggesting that the stability of ApoE isoproteins could be affected by their PTMs. Flowers et al. (2020) reported that in the N-terminal domain, plasma ApoE contained richer sialylated core 1 structures compared to CSF ApoE. However, CSF ApoE held substantially more sialylation than plasma ApoE in the C-terminal domain, an observation that was further validated in a similar analysis by Lawler et al. (2023). The finding that CSF ApoE has a much higher sialylation content than plasma ApoE is particularly important in the context of lipoprotein particle interaction, as previous studies have suggested that the additional negative charge of sialylated glycans may facilitate HDL binding (Marmillot et al., 1999; Huang et al., 2014). Our group first reported that ApoE protein isoforms in the human brain undergo varying degrees of sialylation, with ApoE2 being most abundantly sialylated, whereas ApoE4 is least sialylated. Quantification indicated that the ratio between the sialoforms versus the asialoform was 2.50 for ApoE2, 1.36 for ApoE3, and 0.62 for ApoE4. Our analyses further revealed that the sialic acid moiety expressed on human ApoE protein robustly modulated ApoE interactions with Aβ peptides and Aβ fibrillation (Moon et al., 2022).

## 5 Human ApoE glycosylation and sialylation: physiology and pathophysiology

Despite a limited number of studies, current literature underscores the pivotal role of ApoE glycosylation and sialylation in alterations of lipid and Aβ metabolism. Potential influences of these PTMs on pathophysiology have also been explored to some extent in both peripheral and CNS disorders such as metabolic syndrome, gynecological diseases, and AD (Marmillot et al., 1999; Atkinson et al., 2009; Savinova et al., 2014; Uen et al., 2015). Even though there have been recent advancements, it is imperative to acknowledge that a substantial body of research is still critically required to fully unveil the opportunities of this highly promising yet historically neglected area of research in the ApoE field.

### 5.1 Modulation of lipid metabolism

A major development in the research in understanding the biological role of ApoE glycosylation and sialylation came around 1999 when Marmillot et al. (1999) first reported that the interaction of ApoE with different lipoproteins can be differentially impacted by the sialylation status of ApoE. Specifically, it was found that the binding affinity of ApoE towards HDL decreased significantly in response to the removal of sialic acid moiety on ApoE (11.2-fold decrease), whereas the ApoE association with VLDL was mildly affected (2.7-fold decrease). Additionally, the HDL-binding strength of desialylated ApoE was restored with the re-addition of sialic acid by treatment with a combination of sialyltransferase (ST) and cytidine monophosphate-*N*-acetylneuraminic acid (CMP-NANA), a sialic acid precursor, confirming the significance of sialylation to ApoE association with HDL. The investigators further reported that chronic ethanol exposure disrupts the stability of ST mRNA, which, as a result, decreases the hepatic synthetic rate of ST, resulting in impaired sialylation of ApoE. This impairment of ApoE sialylation weakens the association of ApoE with HDL and consequently hampers the reverse cholesterol transport process facilitated by HDL (Lakshman et al., 1999). Similarly, Ghosh et al. (2000) observed that macrophages isolated from rats that had been fed an alcoholic diet for 8 weeks expressed ApoE with 47.6–67.2% lower HDL_3_ binding capacity; however, this reduced capacity was entirely reversed by the re-sialylation of the desialylated ApoE. Moreover, it was observed that in human monocyte-derived macrophages, treatment with oleic acid, but not palmitic acid, exerted a differential impact on the synthesis of the two most abundant sialylated isoforms of ApoE. Specifically, oleic acid upregulated the synthesis and secretion of the less sialylated isoform of ApoE; however, it downregulated the synthesis and secretion of the more sialylated isoform of ApoE. The underlying mechanism is unknown, although it was speculated that oleic acid-mediated alterations of ApoE sialylation might have involved changes in phospholipid composition that further impacted the conformation and availability of ApoE as a substrate for posttranslational sialylation (Huang et al., 2004). Taken together, the findings from these studies indicate a bidirectional regulatory interplay between ApoE sialylation and lipid metabolism in a specific tissue or cellular environment.

In a cohort study involving patients with metabolic syndrome (MetSyn), diagnosed based on the presence of at least three or more components from the list of central obesity, reduced HDL-cholesterol, elevated plasma triglycerides or glucose, or high blood pressure, ApoE glycosylation in VLDL, LDL, and HDL fractions was observed to be decreased by 17, 30, and 25%, respectively. This finding provides further support for the potentially significant role of the glycosylation status of ApoE in the regulation of the physiological function of different lipoproteins and the pathogenesis of associated metabolic disorders (Savinova et al., 2014). Building on this understanding, Ke et al. (2020) investigated the impact of ApoE glycosylation on the atherogenicity of the plasma LDL L5 subfraction; the percentage of L5 in total LDL has been reported to be significantly higher in patients with MetSyn than in healthy individuals. Their investigation found that ApoE in L1 (least negatively charged with no harmful effects) versus L5 (most negatively charged and pro-atherogenic) subfractions of plasma LDL exhibited distinct glycosylation patterns. ApoE in L5 subfraction was found to be highly glycosylated and sialylated at Ser94, Thr194, and Thr289, all containing a HexNAc-Hex-NeuAc glycan sequence. In stark contrast, no glycosylation was detected on L1 ApoE. Structural analyses predicted that the negatively charged glycans on L5 ApoE may hamper ApoE interaction with LDLR, thereby compromising LDLR-mediated endocytosis and internalization of LDL.

In light of the well-documented findings, including (1) ApoE isoforms are differentially sialylated; (2) ApoE isoforms exhibit distinct specificities in their associations with lipoproteins; and (3) sialylation appears to play a prominent role in altering ApoE interactions with different lipoproteins, it can be speculated that the sialylation status may server as a critical regulator of the differential roles of ApoE isoforms in lipid metabolism. This hypothesis, along with the contributions from specific residues and the regulatory mechanisms, needs to be addressed in future studies.

### 5.2 Modulation of Aβ metabolism

Though Aβ’s role in AD development is still disputed, the formation of Aβ plaques is an established hallmark in AD pathogenesis (Aleshkov et al., 1997; Wisniewski and Drummond, 2020). Many studies have shown that ApoE interacts with Aβ in an isoform-specific manner and that the ApoE-Aβ complex contributes to Aβ metabolism in the brain (Strittmatter et al., 1993b; LaDu et al., 1994; Zhou et al., 1996; Manelli et al., 2004; Yamazaki et al., 2019). While studies have attempted to rationalize the association between ApoE and Aβ and how it might promote Aβ pathogenesis (Holtzman, 2001; Wisniewski and Drummond, 2020), how ApoE isoforms differentially interact with Aβ remains elusive.

ApoE expressed in the CNS is highly glycosylated and sialylated, and the status of these PTMs has been indicated to affect the interaction of ApoE with Aβ, although the results are mixed (Sugano et al., 2008; Moon et al., 2022; Meuret et al., 2023). ApoE *O*-glycosylation was first identified at Thr194 (Wernette-Hammond et al., 1989). A mutation of this glycosylation residue (T194A) in ApoE2 was not found to affect the SDS-stable binding of ApoE to Aβ. It should be pointed out in this study that recombinant ApoE2 was expressed in baby hamster kidney fibroblasts, commonly known as BHK cells (Aleshkov et al., 1999). Contrary to this earlier finding, a later report described that removing glycans by point mutation of T194A in T-Rex 293 cell-derived ApoE3 resulted in decreased binding to Aβ42 compared to wildtype ApoE3. Moreover, neuraminidase treatment abolished the binding avidity of ApoE-containing lipoprotein for Aβ42 (Sugano et al., 2008). In contrast to the Sugano report, a recent study from our group indicated that brain ApoE2 undergoes the most extensive sialic acid modification among the three ApoE protein variants. Our study further demonstrated that removing the sialic acid moiety significantly increased ApoE2 binding affinity with Aβ peptides and promoted Aβ fibrillation. Moreover, we identified the sialic acid moiety for the Aβ17-24 region and promoted Aβ fibrillation (Moon et al., 2022). The Aβ17-24 region is a mid-region in Aβ peptides and has previously been defined as the ApoE-Aβ binding site. Small peptides designed to disrupt ApoE interaction with this ApoE binding region on Aβ were found efficacious in reducing intraneuronal Aβ accumulation and synaptic degeneration (Strittmatter et al., 1993b; Sadowski et al., 2004; Kuszczyk et al., 2013; Pankiewicz et al., 2014; Liu et al., 2017). Taken together, our findings provide a plausible explanation for the well-documented roles of ApoE isoforms in Aβ pathogenesis. Specifically, compared to ApoE3 and ApoE4, the higher expression of sialic acid in ApoE2 may contribute to the less potent interaction between ApoE2 and Aβ, ultimately, the slower rate of brain Aβ deposition, a mechanism thought to underlie ApoE2-mediated decreased risk for AD (Moon et al., 2022). So, what may have contributed to the conflicting results observed in Sugano versus Moon’s studies? One of the contributing factors may be attributed to differences in the ApoE samples analyzed in these two studies. The study by Sugano et al. (2008) involved the analysis of ApoE-containing lipoproteins isolated from human serum. It is pertinent to note that blood-derived lipoproteins are complex molecules comprising a heterogeneous mix of lipids and various apolipoproteins, including ApoA, ApoB, ApoC, and ApoE. In the data reported it was observed that ApoE sialylation remained unchanged even after exposure to neuraminidase (10–100 U/L). Despite this, both SPR and western blot analysis data revealed significant reductions in Aβ binding, strongly suggesting that this observed decrease in Aβ binding did not originate from changes in the sialylation status of ApoE. Overall, the data generated does not support the conclusions drawn by the authors. On the contrary, in the Moon et al. (2022) study, ApoE2 was pure recombinant human protein derived from 293-F cells, and most importantly, its glycosylation and sialylation patterns resembled the physiological form of ApoE2 in the human brain. Apart from the sample differences, the inconsistency observed in the Sugano vs. Moon reports could also be due to the glycosylation sites studied. In the Sugano report, changes in Aβ binding revealed in the cell culture data were explicitly associated with the sialylation status of Thr194. In contrast, in the Moon report, changes in Aβ binding reflected a total effect of the overall sialylation status of ApoE2, suggesting that the sialylation status of other glycosylation sites than Thr194 may have a stronger influence on ApoE interaction with Aβ. Sources of species where ApoE is expressed are another concerning limitation in the current literature, making findings derived from non-human sources less relevant (Chua et al., 2010).

Recent studies have extended the scope of research to patients with cognitive impairment to understand the associations of ApoE with Aβ and how the degree of glycosylation affects the interaction (Lawler et al., 2023; Meuret et al., 2023). CSF ApoE glycosylation was correlated with higher levels of CSF Aβ42 and small HDL particles. ApoE sialylation promoted the degree of Aβ42 degradation in an ApoE isoform-specific manner in microglial cells. In addition, removing sialylation from ApoE3 increased CSF ApoE binding toward heparin (Meuret et al., 2023). The association of ApoE glycosylation and Aβ was further demonstrated in plasma samples collected from a cohort of older individuals with a mean age of 75.6 years and 49% with cognitive impairment. Plasma ApoE glycosylation occupancy in the hinge region was found to be negatively associated with ApoE4 carrier status; however, it was positively associated with amyloid status as determined by CSF Aβ42/Aβ40 (Lawler et al., 2023). These clinical findings provide further support for a potentially significant role of ApoE glycosylation and sialylation in modulating AD-related metabolic processes, which could be translated into a beneficial effect that prevents or delays the onset of dementia.

In summary, based on the current literature, it can reasonably be concluded that ApoE glycosylation and sialylation strongly influence Aβ metabolism. The differential glycosylation and sialylation status of ApoE isoforms serves as a key modulator of ApoE interaction with Aβ, which provides a plausible explanation for the well-established ApoE isoform-dependent Aβ plaques and, ultimately, AD risk. That said, the observed inconsistencies underscore the need for a more nuanced approach in future studies that take into consideration the inherent variability associated with species and tissue and cell types, as discussed above. An additional avenue of crucial research involves an in-depth exploration of the impact of sialylation on ApoE in relation to inflammation within the brain, a hallmark of AD pathology. Siglecs, a family of immunoglobulin-type lectins, are primarily found on the cell surfaces in brain microglia. Acting as pivotal anti-inflammatory mediators, they convey inhibitory signals upon engaging with sialic acid. This interaction effectively deactivates microglia, thereby playing a vital role in controlling neuroinflammatory processes, with ApoE4 associated with reduced capability to suppress inflammatory stimuli (Guo et al., 2004; Dorey et al., 2014; Linnartz-Gerlach et al., 2014; Rawal and Zhao, 2021; Wightman et al., 2021). Understanding how the sialylation structure profile influences ApoE’s role in regulating inflammatory responses may open new opportunities for therapeutic interventions targeting inflammation-associated pathogenic processes in neurodegenerative disorders.

### 5.3 Modulation of disease pathophysiology

In addition to metabolic syndrome and AD associated with lipid and Aβ metabolism, ApoE glycosylation and sialylation may pose an influence on the development of other diseases as well. Preeclampsia is a pregnancy complication that can cause preterm birth and fetal growth restriction. It is characterized by sudden-onset hypertension (after 20 weeks of gestation) and at least one other related complication, including proteinuria, maternal organ dysfunction, or uteroplacental dysfunction (Dimitriadis et al., 2023). An earlier study from Atkinson et al. (2009) reported that ApoE glycosylation profiles in pregnant women with preeclampsia were significantly altered compared with healthy pregnant women. In preeclampsia plasma, there were no changes in total ApoE plasma concentration; however, the level of a deglycosylated form of ApoE was increased, concomitant with the decreased level of a highly sialylated glycoform of ApoE. The authors speculated that these changes in the composition of ApoE glycoforms in the plasma could result in reduced ApoE association with HDL, impairing reverse cholesterol transport and, as a result, increasing the risk for vascular damages.

In a subsequent investigation aimed at expanding the comprehension of the impacts of PTMs in ApoE on the pathogenesis of other women’s diseases of the reproductive system, Uen et al. (2015) conducted a study revealing a correlation between the occurrence of ApoE glycosylation and the susceptibility to breast cancer. The data showed that, consistent with the preeclampsia study, plasma levels of total ApoE did not differ between breast cancer patients and healthy women. However, the study identified a previously unknown *O*-glycosylation site at Ser129 on plasma ApoE, and the degree of glycosylation of this residue was found to be slightly increased in breast cancer samples compared to healthy controls.

Alteration of ApoE glycosylation profiles observed in preeclampsia and breast cancer could indicate that ApoE glycosylation status might also be a distinguishing feature in other gynecological diseases. Further studies focusing on understanding the underlying molecular mechanisms linking ApoE glycosylation to these diseases would contribute to a more comprehensive elucidation of their pathophysiology. Additionally, exploring the clinical relevance and prognostic values could pave the way for personalized diagnostic and therapeutic approaches.

## 6 Conclusions and future perspectives

Human ApoE exists in three major isoforms, ApoE2, ApoE3, and ApoE4, which confer different risks in developing LOAD. Several rare variants of ApoE have also been associated with AD, increasing either brain resilience or risk for AD. Since the discovery of the genetic association of ApoE genotypes with AD in the 90s, intensive research efforts over the last three decades have yielded enormous amounts of knowledge, with over 10,000 papers documenting numerous processes impacted by ApoE. Despite all the advancements, the fundamental question remains regarding the underlying mechanism that gives rise to various effects differed by ApoE genotypes. ApoE is posttranslationally glycosylated in a species-, tissue-, and cell-specific manner. Human ApoE, particularly in brain tissue and CSF, is highly glycosylated, and the glycan chains are exclusively attached via an *O*-linkage to Ser or Thr residues. Eight *O*-linked glycosylation sites in human ApoE have been identified and confirmed by at least two independent reports. Moreover, studies have indicated that human ApoE glycans undergo sialic acid modification or sialylation, a structural alteration more prominent in ApoE derived from the brain and CSF than plasma. Furthermore, the three primary variants of ApoE have recently been revealed to undergo varying degrees of sialylation, with ApoE2 exhibiting the most abundant sialic acid modification, whereas ApoE4 is the least sialylated. The functional significance of ApoE glycosylation and sialylation has been mainly demonstrated in their modulation of ApoE interactions with lipids and Aβ and, consequently, lipid and Aβ metabolism. Overall, despite the limited work in the literature, the available evidence provides compelling support to the notion that ApoE glycosylation and sialylation may serve as an essential modifier that alters not only the structure but also the function leading to the distinct effects of ApoE isoforms in AD and possibly other diseases as well.

Regrettably, despite the fact that ApoE has long been recognized as a highly sialylated glycoprotein, the potential impacts of these PTMs on human health and disease have been largely neglected in the ApoE research field until recently. Many studies have used recombinant human ApoE protein expressed in a prokaryotic system, mainly *E. coli*. However, the *E. coli* expression system lacks the necessary machinery to perform PTMs, producing only the non-PTM form of protein. Thus, findings generated from studies of *E. coli*-derived non-PTM form of ApoE may fail to recapitulate the actual biology of human ApoE. In addition, given the significant disparities in the availability of glycosylation and sialylation residues in ApoE of different species, in particular, considering the nearly non-detectable sialylation of mouse ApoE, using mouse-derived cell or *in vivo* models may also not be suitable for studies of ApoE PTMs. These limitations in the published work may have not only hampered advancements in understanding the biology of ApoE PTMs but also inevitably contributed to disparate outcomes observed in different studies. Therefore, using appropriate models and sample sources of human ApoE is the first and most crucial requirement for research to elucidate the structural diversity and corresponding functional roles of ApoE PTMs, such as glycosylation and sialylation, in human health and diseases, including AD.

An additional source of the observed discrepancies within the existing body of literature could be from the glycosylation site analyzed in specific studies. For a long time, it was believed there was only one *O*-glycosylation site at Thr194 on human ApoE. Accordingly, earlier studies had mainly focused on the role of the glycosylation and sialylation status of Thr194. In recent years, advancements in the development of methodologies have significantly increased the sensitivity in detecting less abundant glycan structures, leading to the identification of up to eight *O*-glycosylation sites on human ApoE. Research has indicated that these sites can have different expressions of glycosylation and sialylation profiles within the same tissue or cellular environment, and the same sites can also exhibit divergent profiles in different tissue or cellular environments. This diversity is further expanded by the differences observed among ApoE isoforms. Therefore, investigations designed to elucidate differential glycosylation and sialylation properties of ApoE isoforms within specific tissue or cell types will be essential for gaining insights into the localized roles of these PTMs on ApoE-related physiology and pathophysiology.

In conclusion, recent research has highlighted an emerging and exciting opportunity in the quest for an ultimate answer to the long-standing question in the ApoE research field. The glycosylation and sialylation status of ApoE could potentially serve as a prognostic biomarker for predicting AD risk; it could also serve as a potential therapeutic target for preventing or ameliorating the formation of AD-related amyloidosis and other pathologies. Future research needs to continue to solidify and expand the current state of knowledge, address the underlying mechanisms leading to isoform-specific profiles, and evaluate whether enhancing ApoE4 glycosylation and sialylation would improve the function of ApoE4 in modulating cellular processes and ultimately increase the ApoE4 brain resilience against AD and possibly diseases of the periphery as well.

## Author contributions

H-JM: Writing – original draft, Writing – review & editing. YL: Writing – original draft, Writing – review & editing. DC: Writing – original draft, Writing – review & editing. LZ: Writing – original draft, Writing – review & editing.
